# The Grady Hospital

**Published:** 1891-02

**Authors:** 


### THE GRADY HOSPITAL.

For a number of years the JOURNAL has advocated the establishment of a first-class hospital in Atlanta. And for so many years have no steps been taken towards this end. It remained for the death of the man, who built so many noble monuments to his name during his life, to be the incentive leading those who have begun the work to erect a monument to his name which shall be more enduring than bronze or marble statues. It is with feelings of deepest pleasure that we have seen the corner-stone of the Grady Hospital laid and the walls begin to rise above their foundations. The future of this institution is assured, and the good it shall do is incalculable. Let it become to this portion of the South what similar institutions in New York and Philadelphia are to their section of our country—a home for the destitute sick and a hall of instruction for the numerous students who come here to learn the healing art.

In the beginning of this work Atlanta has taken another step above the level of a little town. That all needed support and all interest in its success shall not be lacking we cannot doubt. The name it bears and the good it can do will suffice as an "*open sesame*" to the hearts and purses of an ever generous people. Let its internal management follow the model of the best hospitals in this country or abroad, and may the day never come when it shall be the lever of power to advance selfish interests or ambitions. The JOURNAL expects to always be one of the best friends of the Grady Hospital.

